# Patient Engagement in a Mobile App–Based Rehabilitation Program for Total Hip or Knee Arthroplasty: Secondary Data Analysis of a Randomized Controlled Trial

**DOI:** 10.2196/57635

**Published:** 2024-10-01

**Authors:** Qingling Wang, Regina Lai-Tong Lee, Sharyn Hunter, Aiyong Zhu, Sally Wai-Chi Chan

**Affiliations:** 1 School of Nursing and Health Management Shanghai University of Medicine and Health Sciences Shanghai China; 2 School of Nursing and Midwifery College of Health, Medicine and Wellbeing University of Newcastle Newcastle Australia; 3 The Nethersole School of Nursing Faculty of Medicine The Chinese University of Hong Kong Hong Kong China (Hong Kong); 4 Tung Wah College Hong Kong China (Hong Kong)

**Keywords:** total hip arthroplasty, total knee arthroplasty, rehabilitation, mobile health, social media application, patient engagement

## Abstract

**Background:**

Health care professionals use mobile apps to support patients’ rehabilitation after total hip or knee arthroplasty. Understanding patient engagement in such mobile health interventions can help tailor these interventions to better support patients.

**Objective:**

This study aimed to investigate patient engagement in a mobile app–based arthroplasty rehabilitation program and to investigate the association between patient engagement and their characteristics.

**Methods:**

Data were extracted from a pool of 42 participants in the experimental arm of a randomized controlled trial that used a mobile app (WeChat [Tencent Holdings Limited])–based program to support patients’ rehabilitation after total hip or knee arthroplasty. The primary outcomes were the number of days the participants accessed the program and completed recommended rehabilitation tasks. Secondary outcomes included data on the participants’ posts on a discussion forum, messages sent by the participants, access to the program components, and reading and sharing the program content. Generalized linear models were used to analyze the association between patient engagement and personal characteristics.

**Results:**

The participants reported in a rehabilitation diary accessing the program on a mean of 5.2 (SD 2) days per week and completing recommended rehabilitation tasks on a mean of 6.5 (SD 0.8) days per week. The majority (31/42, 74%) posted on the discussion forum, with a mean of 18.1 (SD 21.2) posts. Most participants (37/42, 88%) sent messages to health care professionals, with a mean of 14 (SD 15.9) messages. The program components were visited for a total of 525 times. The program content was read 898 times and shared 82 times in total. Generalized linear models showed that both primary outcomes, the number of days the participants accessed the program (B=6.46, 95% CI 1.98-15.35; *χ*^2^_1_=11.1, *P*=.001) and the number of days they completed rehabilitation tasks (B=2.65, 95% CI 0.45-5.48; *χ*^2^_1_=5.7, *P*=.02), were positively associated with having a high school education or above. In addition, the number of posts on the discussion forum was positively associated with living with family, having a high school education or above, undergoing total knee arthroplasty, having comorbidities, and the score of self-efficacy but was negatively associated with age. The number of messages sent by the participants was positively associated with having a high school education or above, having comorbidities, and the score of self-efficacy.

**Conclusions:**

Patient engagement in mobile arthroplasty rehabilitation is associated with their education level, cohabitation status, age, type of surgery, presence of comorbidities, and sense of self-efficacy. Program developers can consider these characteristics and use strategies, such as family involvement, in the design of mobile arthroplasty rehabilitation programs to enhance patient engagement in such interventions.

**Trial Registration:**

Australian New Zealand Clinical Trials Registry ACTRN12621000867897; https://tinyurl.com/mtdw25fp

## Introduction

Over 3 million total hip arthroplasty (THA) and total knee arthroplasty (TKA) procedures are performed globally each year [1]. Patients after THA and TKA require rehabilitation to relieve pain, enhance muscle strength, improve mobility, optimize functional outcomes such as walking, and improve health-related quality of life [2,3]. However, patients may have difficulty accessing face-to-face rehabilitation services after arthroplasty due to the geographic distance from rehabilitation facilities or lack of local health resources [4]. This is particularly true in China due to the lack of rehabilitation facilities and qualified health care professionals [5]. With technological advancement, health care professionals can use information and communication technologies, such as mobile apps, to complement or as an alternative to face-to-face services, to support patients’ rehabilitation after THA and TKA [6]. Studies have investigated the effectiveness of using mobile apps to support patients’ rehabilitation after arthroplasty and reported positive effects of app-based rehabilitation on health outcomes such as pain, range of motion, physical function, and health-related quality of life [7,8]. However, more evidence from robust randomized controlled trials based on physical and psychological outcomes is needed to support the implementation of mobile rehabilitation in arthroplasty populations [9].

Mobile apps used to provide arthroplasty rehabilitation interventions include those specifically designed for target populations [7], commercial medical apps [10,11], and social media apps such as WeChat (Tencent Holdings Limited) [8]. Social media apps are used by many health care providers due to their high penetration, good interactivity, ease of use, and low cost [12]. In China, WeChat is the most popular free social media platform [13] and has been used to provide health interventions, such as weight control and hypertension management [14,15]. We developed a mobile app (WeChat)–based program that was based on theories [16,17] and patients’ perceived needs [18] to support patients’ rehabilitation in 6 weeks after THA and TKA. A randomized controlled trial was conducted to investigate the effectiveness of the program, and the results showed that the program improved patients’ self-efficacy, physical function, and health-related quality of life and reduced levels of anxiety and depression [19]. Study participants reported that the program facilitated their access to health care, encouraged their postoperative recovery, supported the relationships with health care professionals and other patients in the program, and facilitated their learning during postarthroplasty rehabilitation [20]. However, the extent to which patients were engaged in the mobile app–based rehabilitation program and how this engagement was associated with patients’ characteristics were not investigated.

It is important to understand patient engagement in mobile health interventions because interventions are more efficient and effective when patients are better engaged [21]. In mobile health, patient engagement is defined as “...the ability for apps to enable collaboration, activation and participation, information-sharing, and decision-making in one’s own health” [22]. Patient engagement in mobile health is often indicated by the continued use of an app; the intensity of using a function of the app; information dissemination through the app, such as reading and sharing content; and patients’ performance, such as reactions and comments on posts in a social media app [23-25]. Collection and analysis of data on patient engagement will facilitate the understanding of patients’ preferences for using mobile health programs, disparities among populations, and barriers to engagement, which will further inform the improvements in the design of mobile health programs as well as strategies to enhance patient engagement in mobile health [24,25].

Patient engagement in mobile arthroplasty rehabilitation has not been extensively explored. Previous studies analyzed usage data (eg, log-in frequency) automatically recorded by commercial medical apps [11] or apps specifically designed for arthroplasty populations [7,26] and reported that 11% to 14% of patients did not adhere to the mobile rehabilitation programs recommended by health care professionals. One reason was that patients could not interact with the program because of technical challenges, such as the need to set up sensors and touch the device multiple times to start a session [7,26]. However, a research gap exists in patient engagement in social media–based health interventions, requiring further studies to explore engagement assessment as well as strategies to promote patient engagement [27]. For example, measuring patient engagement in WeChat-based interventions may differ from that based on specifically designed or commercial medical apps that could track log-in data, as WeChat’s privacy policy does not allow researchers to access individual data on log-in frequency and duration [28]. A self-reported rehabilitation diary and counting data (eg, number of posts and messages) may provide information about patient engagement in such social media app–based programs [29].

Furthermore, evidence of the association between patient engagement in mobile health and personal characteristics is inconclusive. For example, a study on a mobile program to support medication adherence reported that age was positively associated with the duration of patients’ usage of the program, while gender and education level had no association with either duration or intensity of usage [30]. Another study found that age, education level, family monthly income, and employment status were associated with the usage duration and log-in frequency in a mobile breast cancer support program [31]. In mobile arthroplasty rehabilitation, patient engagement may be influenced by factors such as age, pain, and limited physical function after surgery [32,33]. In addition, surgical procedures, such as THA and TKA, may also affect patients’ use of social media for health purposes [34]. Elucidating the association between patient engagement and their characteristics can guide the development of programs that are delivered through social media platforms to make them acceptable to patients and thus effective in supporting patients’ rehabilitation [35].

This study aimed to investigate patient engagement in a social media app–based rehabilitation program for THA and TKA by analyzing data that had been collected in a randomized controlled trial. The objectives were to (1) investigate patient engagement in the 6-week mobile arthroplasty rehabilitation program through data collected from a written rehabilitation diary, data on the patient’s performance in the program, and data tracked by the app’s background system; and (2) examine the association between patient engagement and their demographic and clinical characteristics.

## Methods

### Study Participants

This study extracted data from the pool of 43 participants in the experimental arm of a single-center, parallel-group, randomized controlled trial conducted at a university hospital in Shanghai, China. All participants were adults (aged ≥18 years) who (1) were discharged home after a unilateral primary THA or TKA and (2) had access to a 6-week, mobile app–based rehabilitation program after hospital discharge. Patients were excluded from this study if they could not practice recommended rehabilitation exercises due to major complications or serious health conditions such as heart failure or had severe vision impairment.

### Description of the Mobile App–Based Rehabilitation Program

A 6-week, theory-underpinned rehabilitation program was provided to study participants through the app WeChat, aiming to enhance their self-efficacy during rehabilitation and improve their rehabilitation practice and outcomes. The program provided exercise demonstration videos that were designed for patients after THA and TKA. The participants were suggested to follow the videos and practice for 1 hour per day and at least 5 days per week. In addition, program components were designed to enhance patients’ self-efficacy for rehabilitation, such as setting weekly goals for rehabilitation; scheduling progressive rehabilitation tasks; providing short stories and videos to share the experiences of previous patients; holding a discussion forum that involved health care professionals, patients, and researchers through the app; and providing psychological techniques such as relaxation exercises. To facilitate the participants’ learning process, educational materials in the program were designed using visual presentations with short text messages, and a paper booklet on the use of the program was provided in addition to verbal explanations. A detailed description of the program has been published [36].

The mobile app–based rehabilitation program was carried out between May 2021 and January 2022. Educational materials such as demonstration videos were uploaded to the app (WeChat) in advance. The participants could learn at home through WeChat installed on their own mobile devices. To improve participants’ engagement in the program, the researchers sent an exercise reminder every Monday, and the participants were encouraged to record their usage of the program and completion of recommended rehabilitation tasks in a written rehabilitation diary.

### Patient Engagement in the Program

Patient engagement in the mobile rehabilitation program was evaluated using 3 measures. The first measured the number of days the participants accessed the program and the number of days the participants completed recommended rehabilitation tasks. These data were obtained by reviewing the participants’ records in their rehabilitation diary. The total number of days and the number of days per week were calculated for each participant. The second measure counted the number of participants’ posts on the discussion forum and the number of messages sent by the participants to health care professionals. The percentages of participants posting on the discussion forum and sending messages were calculated, as well as the number of posts and messages for each participant. The third measure focused on the data from the background system of the app, which tracked how many times the content from the program was read and shared by the participants and how often the program components were visited.

The primary outcomes of this study were the number of days the participants accessed the program and the number of days they completed the recommended rehabilitation tasks as recorded in the paper diary. These outcomes reflected individual engagement in the mobile rehabilitation program. Secondary outcomes focused on engagement in a part of the program, including the number of posts made by the participants on the discussion forum, the number of messages they sent to health care professionals through the app, the total number of visits to the program components, and the total number of times the content was read and shared.

### Demographic and Clinical Characteristics

Before starting the mobile rehabilitation program, study participants completed a paper questionnaire that collected their demographic and clinical characteristics. Demographic data included age, gender, BMI, residence, cohabitation status, education level, and employment. Clinical data included the type of surgery; reasons for surgery; comorbidities; long-term use of medication; and health assessment outcomes such as self-efficacy, self-reported physical function, severity of pain, levels of anxiety and depression, and health-related quality of life. Self-efficacy was measured using the Chinese version of the Self-Efficacy for Rehabilitation Outcome Scale, a 12-item scale that measures the patient’s belief in their ability to perform physical rehabilitation behaviors after hip or knee surgery [37,38]. Patient-reported physical function was measured using the Chinese version of the Hip Disability and Osteoarthritis Outcome Score Physical Function Short Form for THA and the Knee Injury and Osteoarthritis Outcome Score Physical Function Short Form for TKA [39,40]. The severity of pain was measured using the Numeric Rating Scale for Pain [41]. Levels of anxiety and depression were measured using the Chinese version of the Hospital Anxiety and Depression Scale [42]. The patient’s health-related quality of life was measured using the Chinese version of the 5-level EQ-5D version developed by the EuroQol Group [43].

### Statistical Analysis

Data were analyzed using IBM SPSS Statistics (version 28.0; IBM Corp) [44]. Numerical data were described using means with SDs and medians with IQRs. Categorical data were described using frequencies and percentages. In association analyses, data on the number of days accessing the program and the number of days completing rehabilitation tasks were categorized as “equal to or greater than 30 days” and “less than 30 days or missing data” since the participants had been suggested to practice rehabilitation at least 5 days per week (30 days in total) with the support from the program.

As the outcome variables were categorical data (eg, 2 categories of the number of days accessing the program) or counting data (eg, the number of posts and messages), generalized linear models were used to analyze the association between patient engagement and their demographic and clinical characteristics. Pearson correlation analysis was performed for explanatory variables, and pairs of variables with correlation coefficients greater than 0.7 (age and employment as well as comorbidities and long-term use of medication) were discussed within the research team. A decision was made to remove 2 variables, employment and long-term use of medication, from the model, as they might be due to highly correlated variables. A collinearity diagnostic was conducted for the remaining explanatory variables. The results showed that the tolerance of the variables was greater than 0.2 and the variance inflation factor was less than 5, indicating that there was no significant collinearity between the variables. Therefore, all remaining explanatory variables (ie, age, gender, BMI, residence, cohabitation status, education level, type of surgery, reasons for surgery, comorbidities, self-efficacy, self-reported physical function, severity of pain, levels of anxiety and depression, and health-related quality of life) were included in generalized linear models. Considering the coprimary outcomes, the statistical significance was set as an α level of .025.

### Ethical Considerations

Ethical approval to conduct this study was obtained from the Human Research Ethics Committee of the University of Newcastle in Australia (reference H-2020-0414) and from the study hospital (reference B2021-096R). All participants signed a written informed consent before they were enrolled in the study. The original approval covers secondary data analysis without additional consent.

## Results

### Participant Characteristics

As 1 patient dropped out from the study within 1 week after group allocation and did not participate in the mobile rehabilitation program, this study included 42 participants in total. Women comprised 74% (31/42) of the participants, and the age ranged from 33 to 83 (mean 66.7, SD 10.4) years. The participants had undergone THA (23/42, 55%) or TKA (19/42, 45%). Most participants underwent surgery because of osteoarthritis (35/42, 83%), followed by acute fractures (5/42, 12%) and other reasons (2/42, 5%). The majority (30/42, 71%) had chronic diseases, such as hypertension (19/42, 45%) and diabetes mellitus (9/42, 21%), and were taking medication for these diseases. Most participants (39/42, 93%) lived with their families after THA and TKA surgery, and 90% (38/42) of the participants were unemployed or retired at the time of the study. In terms of education levels, 24% (10/42) had completed education at university or college, 31% (13/42) had completed high school education or had equivalent learning experience, 29% (12/42) had completed middle school education, and 17% (7/42) had primary school education or lower.

### Patient Engagement in the Mobile App–Based Rehabilitation Program

The majority of study participants (26/42, 62%) returned their completed rehabilitation diary to health care professionals. In the diaries, 65% (17/26) reported accessing the program in 30 or more days, and 96% (25/26) reported completing recommended rehabilitation tasks in 30 or more days. The number of days that the participants accessed the rehabilitation program ranged from 1.3 to 7 (mean 5.2, SD 2; median 5.8, IQR 4.1) days per week, and the number of days that the participants completed recommended rehabilitation tasks ranged from 3.5 to 7 (mean 6.5, SD 0.8; median 7, IQR 0.8) days per week. Table 1 presents descriptive statistics on participant-reported access to the program and completion of rehabilitation tasks, and an exploratory analysis of changes in these data over 6 weeks is provided in Multimedia Appendix 1.

As shown in Table 1, the majority (31/42, 74%) posted questions or comments on the discussion forum in the app. The number of posts ranged from 1 to 70 (mean 18.1, SD 21.2; median 10, IQR 22). Most participants (37/42, 88%) sent messages to health care professionals. The number of messages ranged from 1 to 69 (mean 14, SD 15.9; median 10, IQR 11). The app’s background system showed that the participants read the educational materials in the program 898 times, with a mean of 21.4 times per person, and shared these materials 82 times, with a mean of 2 times per person. The most frequently visited program components were related to the rehabilitation after arthroplasty, such as exercise demonstration videos (243/525, 46%), precautions after the surgery (100/525, 19%), and follow-up schedules (84/525, 16%). They are followed by the stories and short videos shared by previous patients (55/525, 11%) and psychological support components (43/525, 8%).

**Table 1:** Patient engagement in the mobile app–based rehabilitation program for total hip or knee arthroplasty during the 6-week intervention.

Patient engagement in the program	Value, min^a^	Value, max^b^	Value, mean (SD; 95% CI)	Value, median (IQR; 95% CI)
Number of days accessing the program (n=26; d/wk)	1.3	7	5.2 (2; 4.4-6.0)	5.8 (4.1; 4.4-6.9)
Number of days completing rehabilitation tasks (n=26; d/wk)	3.5	7	6.5 (0.8; 6.2-6.8)	7 (0.8; 6.7-7.0)
Number of posts on the discussion forum (n=31; times)	1	70	18.1 (21.2; 10.4-25.9)	10 (22; 5-16)
Number of messages sent by the participants (n=37; times)	1	69	14 (15.9; 8.7-19.3)	10 (11; 5-14)

^a^Min: minimum.

^b^Max: maximum.

### Association Between Patient Engagement, Demographic, and Clinical Characteristics

Table 2 shows the association between patient engagement in the mobile app–based rehabilitation program and their demographic and clinical characteristics. The number of days that the participants reported accessing the program was positively associated with having a high school education or above (B=6.46, 95% CI 1.98-15.35; *χ*^2^_1_=11.1, *P*=.001). The number of days that the participants reported completing rehabilitation tasks was positively associated with having a high school education or above (B=2.65, 95% CI 0.45-5.48; *χ*^2^_1_=5.7, *P*=.02). The number of posts that the participants posted on the discussion forum was negatively associated with age (B=–0.09, 95% CI –0.16 to –0.02; *χ*^2^_1_=6.6, *P*=.01) but positively associated with living with family (B=2.74, 95% CI 1.42-4.06; *χ*^2^_1_=16.6, *P*<.001), having a high school education or above (B=2.46, 95% CI 1.22-3.69; *χ*^2^_1_=15.1, *P*<.001), undergoing TKA surgery (B=1.34, 95% CI 0.35-2.33; *χ*^2^_1_=7, *P*=.008), having comorbidities (B=2.13, 95% CI 0.32-3.95; *χ*^2^_1_=5.3, *P*=.02), and the score of self-efficacy (B=0.33, 95% CI 0.13-0.53; *χ*^2^_1_=10.2, *P*=.001). The number of messages sent by the participants was positively associated with having a high school education or above (B=0.90, 95% CI 0.18-1.62; *χ*^2^_1_=6, *P*=.01), having comorbidities (B=1.21, 95% CI 0.20-2.23; *χ*^2^_1_=5.5, *P*=.02), and the score of self-efficacy (B=0.22, 95% CI 0.05-0.39; *χ*^2^_1_=6.2, *P*=.01).

**Table 2:** Associations between patient engagement in the mobile app–based rehabilitation program and demographic and clinical characteristics (n=42).

Variables		Number of days accessing the program^a,b^			Number of days completing rehabilitation tasks^a,c^			Number of posts on the discussion forum			Number of messages sent by the participants	
		Chi-square (*df*)	*P* value	Chi-square (*df*)		*P* value	Chi-square (*df*)		*P* value	Chi-square (*df*)		*P* value
Age		0.1 (1)	.72	0.1 (1)		.75	6.6 (1)		.01^d^	4.5 (1)		.03
Gender^a,e^		2.2 (1)	.14	0.3 (1)		.60	0 (1)		.99	0.8 (1)		.38
BMI		0.1 (1)	.75	2.4 (1)		.12	1.2 (1)		.27	0 (1)		.97
Residence^a,f^		0.2 (1)	.68	0.1 (1)		.75	0.1 (1)		.72	0 (1)		.98
Cohabitation status^a,g^		0.5 (1)	.47	0 (1)		.84	16.6 (1)		<.001^d^	3.6 (1)		.06
Education level^a,h^		11.1 (1)	.001^d^	5.7 (1)		.02^d^	15.1 (1)		<.001^d^	6 (1)		.01^d^
Types of surgery^a,i^		1.2 (1)	.28	0.1 (1)		.72	7 (1)		.008^d^	0.3 (1)		.57
Reasons for surgery^a,j^		1.8 (1)	.18	3.6 (1)		.06	0 (1)		.99	4.3 (1)		.04
Comorbidities^a,k^		2.7 (1)	.10	0.3 (1)		.61	5.3 (1)		.02^d^	5.5 (1)		.02^d^
**Health outcomes**												
	SER^l^	0.1 (1)	.75	0 (1)		.99	10.2 (1)		.001^d^	6.2 (1)		.01^d^
	HOOS-PS^m^ or KOOS-PS^n^	4.7 (1)	.03	0.1 (1)		.71	0.1 (1)		.71	0.5 (1)		.47
	NRS^o^ Pain	1 (1)	.32	0 (1)		.99	4.2 (1)		.04	4 (1)		.05
	HADS^p^-anxiety	4.2 (1)	.04	0 (1)		.94	0 (1)		.94	0.4 (1)		.52
	HADS-depression	2.3 (1)	.13	0 (1)		.99	0.8 (1)		.37	4.6 (1)		.03
	EQ-5D index	4.5 (1)	.03	2.2 (1)		.14	0.5 (1)		.46	1.4 (1)		.23
	EQ-VAS^q^	4.4 (1)	.04	1.4 (1)		.24	3.6 (1)		.06	1.7 (1)		.19

^a^Grouped into categories. Ascending category order was used in data analysis.

^b^Number of days accessing the program: “1=equal to or greater than 30 days” and “2=less than 30 days or missing data.”

^c^Number of days completing rehabilitation tasks: “1=equal to or greater than 30 days” and “2=less than 30 days or missing data.”

^d^Significant at an α level of .025.

^e^Gender: “1=men” and “2=women.”

^f^Residence: “1=in Shanghai” and “2=in other cities.”

^g^Cohabitation status: “1=living with family” and “2=living alone.”

^h^Education level: “1=high school and higher” and “2=middle school and lower.”

^i^Types of surgery: “1=total knee arthroplasty” and “2=total hip arthroplasty.”

^j^Reasons for surgery: “1=osteoarthritis” and “2=other reasons.”

^k^Comorbidities: “1=yes” and “2=none.”

^l^SER: Self-Efficacy for Rehabilitation Outcome Scale.

^m^HOOS-PS: Hip Disability and Osteoarthritis Outcome Score Physical Function Short Form.

^n^KOOS-PS: Knee Injury and Osteoarthritis Outcome Score Physical Function Short Form.

^o^NRS: Numeric Rating Scale.

^p^HADS: Hospital Anxiety and Depression Scale.

^q^VAS: Visual Analogue Scale.

## Discussion

### Principal Findings

This study investigated patient engagement in a social media app (WeChat)–based arthroplasty rehabilitation program by analyzing data obtained from the app (eg, posts, messages, and reading and sharing of program content) and a written rehabilitation diary (eg, days patients accessed the program and completed recommended rehabilitation tasks). The proportions of patients who accessed the WeChat–based program and who completed recommended rehabilitation tasks are comparable with that reported in previous arthroplasty rehabilitation programs using specifically designed apps or commercial medical apps [7,11,26]. Usage data, such as the number of days per week the patients accessed the program, the average number of posts on the discussion forum, and the average number of messages sent by the patients, are also similar to that in a TKA rehabilitation program based on a commercial medical app [10]. The previous programs were considered to have high levels of patient acceptance and engagement [10,26]. These findings provide evidence that arthroplasty rehabilitation delivered by WeChat has good patient engagement. Furthermore, the findings from this study suggest that patient engagement in mobile arthroplasty rehabilitation is associated with demographic characteristics such as age, education level, and cohabitation status, as well as clinical characteristics such as type of surgery, presence of comorbidities, and sense of self-efficacy. This adds to the body of evidence about the relationship between patient engagement in mobile arthroplasty rehabilitation and personal characteristics.

Education level was found positively associated with all 4 indicators of patient engagement in this study. This supports the evidence that education levels influence patients’ usage of mobile health technologies. A previous study reported that women with higher levels of education had more frequent log-ins and longer usage durations in an app–based breast cancer support program [31]. The authors explained that this was because women with higher levels of education were more receptive to new knowledge and had a greater capacity for understanding health information. In addition to these reasons, patients with higher education levels may be more familiar with the access and use of mobile health technologies, which may have facilitated the engagement of this group of patients in our study. Conversely, patients with lower education levels may need support to facilitate engagement in mobile health care. Program developers could consider using visual presentations, such as videos and pictures, instead of written materials, and plain language instead of medical jargon to improve engagement in mobile health interventions in populations with lower levels of education.

This study found that patients who lived with their families after surgery posted more frequently on the discussion forum held through the app. Because of cultural contexts such as filial piety and kinship, patients after arthroplasty in China tend to live with their families. Family members are usually the primary caregivers of the patients, participating in and influencing the patient’s decisions about treatment and care [45]. In our study, family members who lived with the patients may have aided the patients’ usage of the mobile app–based rehabilitation program, such as technical assistance, which may have facilitated patient engagement in the program. Patients who have experienced mobile app–based arthroplasty rehabilitation reported that they perceived involving family members would help them better use the programs [20]. Family involvement is also considered important for postarthroplasty recovery in other cultural contexts. One study conducted in 6 European countries recommended the inclusion of family members in health education to fulfill their knowledge expectations and better support patients’ postarthroplasty rehabilitation [46]. Therefore, we recommend including family members in mobile arthroplasty rehabilitation programs, such as giving family members access to health information provided in the program and enabling their involvement in discussions. This will help family members understand arthroplasty rehabilitation and support the patients to be more engaged in the rehabilitation program.

In terms of type of surgery, this study found that patients after TKA were more engaged in the discussion forum than patients after THA. It has been reported that THA procedures offer patients better outcomes on pain relief, daily activity performance, expectations met, and overall satisfaction with the surgery than TKA [47]. Approximately 10% (2,754/25,012) of patients undergoing TKA are dissatisfied because of factors such as unmet expectations and persistent pain [48]. Patients after TKA thus may have more concerns and questions than those after THA about their postarthroplasty recovery and need more support during rehabilitation. The discussion forum of the current program enabled patients to obtain timely support from health care professionals and fellow patients undergoing similar procedures. When investigating the effectiveness of the mobile rehabilitation program, this support was confirmed by the patients [20]. Future studies could investigate the specific support required by patients after TKA and solutions to address the requirements so that mobile rehabilitation interventions are tailored for this population.

In our study, patients with comorbidities, such as hypertension and diabetes mellitus, were more engaged in the discussion forum and sending messages. Patients with such chronic diseases may have developed skills to access health information and support and embraced technology to help them understand and manage their chronic conditions [30,49]. These skills may have facilitated their engagement in the current mobile rehabilitation program for arthroplasty. For instance, in a previous study, a patient who underwent THA with systemic lupus erythematosus said that they were familiar with seeking health information about their condition from the internet, but they preferred to use the mobile rehabilitation program that was specifically designed for patients undergoing arthroplasty as they found that the health information provided by health care professionals in the program was more comprehensive and reliable than that available on the internet [20].

Patients’ self-efficacy scores were found positively associated with the number of posts they posted on the discussion forum and the number of messages they sent to health care professionals. A systematic review reported that self-efficacy as a predictor of patient engagement in online health interventions was inconclusive, as few studies had explored the relationship [50]. Theoretically, patients with a higher sense of self-efficacy are more confident in accomplishing rehabilitation tasks [16]. These patients may be more inclined to view difficulties arising during rehabilitation as challenges that they should put more effort into or seek support from health care professionals and others [51]. In this study, patients were provided with the opportunity to obtain professional and peer support through the discussion forum held through WeChat and sending messages to health care professionals. This may explain why patients with higher self-efficacy scores were more engaged in these 2 components of the program. Future mobile rehabilitation programs could consider identifying patients with a lower sense of self-efficacy and providing support to facilitate their engagement in the program. The theory of self-efficacy [16] could be used to guide the individualization of mobile rehabilitation interventions.

However, this study found that patient engagement, such as posting on the discussion forum, was negatively associated with age. This is consistent with the findings of a previous systematic review that older age is associated with decreased engagement in mobile technology–based monitoring for TKA [32]. Physiological declines with advancing age, such as decreased visual perception and hearing loss, may hinder using mobile technology–based health interventions in older populations [52]. Low technology proficiency was reported as a barrier to engagement in eHealth in older adults [53], but other researchers have also claimed that advanced age is not a major issue in the use of mobile health technologies if adequate orientation and coaching are provided [54]. Zhu et al [31] found that older women were even more engaged in a mobile app–based breast cancer support program than younger women. The authors believed that user-centered design, technical support, and provision of easy-to-understand information would facilitate older patients’ engagement in mobile health interventions [31]. Therefore, age as a predictor of patient engagement in mobile health is inconclusive, and more research is needed to understand the relationship between them and possible mediating factors.

### Study Limitations

This study has limitations. Engagement data were extracted from 1 arm of a randomized controlled trial. This may have introduced selection bias in the results as patients who voluntarily participated in the trial might be more familiar with technology and thus better engaged in the program. The results of this study should be interpreted with caution, as the sample size was small, which may have reduced the power of the statistical analysis [55]. Due to the privacy policy of WeChat, the engagement data from the app’s background system, such as the times of reading and sharing the program content, are global counts, and the data of each participant cannot be identified. This limits the exploration of individual disparities in program usage. The current program is a 6-week mobile rehabilitation program focusing on acute rehabilitation after arthroplasty, which limits the exploration of how patient engagement in mobile rehabilitation changes with recovery after surgery.

Data collected through the written rehabilitation diary may not fully represent patients’ access to and completion of the rehabilitation program. The return rate of the rehabilitation diary was 62% in this study, which is in the middle of the range reported in previous studies where 35% to 86% of patients undergoing THA or TKA returned a written diary to health care professionals [56,57]. However, a large proportion (74% and 88%, respectively) of patients in our study posted and sent messages in the mobile rehabilitation program, suggesting some patients who did not return the diary engaged in the program. App-based electronic diaries have reported high completion rates. For example, a study had 89% to 94% of patients completing the diary [58]. Electronic diaries facilitate the return rate by sending reminders, tracking reports in a real-time way, and collecting data remotely without physical space restriction [59]. However, further investigation is needed on how to incorporate electronic diaries into social media app–based arthroplasty rehabilitation programs. Furthermore, the validity of the written rehabilitation diary may have been reduced if the patients back- and forward-filled in their diaries to meet the expectations of health care professionals [60].

### Conclusions

The social media app–based arthroplasty rehabilitation program can support patient engagement. Patient engagement in mobile arthroplasty rehabilitation is associated with their characteristics, such as education level, cohabitation status, age, type of surgery, presence of comorbidities, and sense of self-efficacy. The future design of mobile app–based arthroplasty rehabilitation programs could consider these characteristics and use strategies, such as involving family members, to improve patient engagement with these interventions.

## Appendix

Multimedia Appendix 1 Analysis of changes in patient engagement in the mobile arthroplasty rehabilitation program over 6 weeks after hospital discharge.

Multimedia Appendix 2 CONSORT-eHEALTH checklist (V 1.6.1).

## Abbreviations

THA: total hip arthroplasty
TKA: total knee arthroplasty
