# Supplementary material for: Patient Engagement in a Mobile App–Based Rehabilitation Program for Total Hip or Knee Arthroplasty: Secondary Data Analysis of a Randomized Controlled Trial
Source: JMIR Mhealth Uhealth. 2024 Oct 1;12:e57635. doi: 10.2196/57635 (PMC11480718; doi:10.2196/57635)
Supplement: Multimedia Appendix 1 [file mhealth_v12i1e57635_app1.docx]

The number of days that the participants reported in their diaries accessing the mobile rehabilitation programme and completing recommended rehabilitation tasks was calculated for each week. Means with standard deviations (SD) are presented in Table 1, and the trend of change is presented in Figure 1.

The number of days that the participants reported accessing the mobile rehabilitation programme reached the peak of 6.0 (standard deviation = 1.9) days at the second week postdischarge, and it slightly decreased to 4.2 (standard deviation = 3.3) days at the sixth week after hospital discharge. The number of days that the participants reported completing recommended rehabilitation tasks is constantly around 6 to 7 days per week during the 6 weeks period.

**Table S1.** Patient engagement in a mobile rehabilitation programme over time (n = 26)

|  | Number of days patients engaged in the programme, mean (SD) | | | | | |
| --- | --- | --- | --- | --- | --- | --- |
|  | Week 1 | Week 2 | Week 3 | Week 4 | Week 5 | Week 6 |
| Accessing the programme | 5.4 (2.5) | 6.0 (1.9) | 5.3 (2.7) | 5.2 (2.9) | 5.2 (2.9) | 4.2 (3.3) |
| Completing rehabilitation tasks | 6.2 (1.8) | 6.7 (0.8) | 6.7 (0.9) | 6.6 (1.2) | 6.8 (0.6) | 5.9 (2.3) |


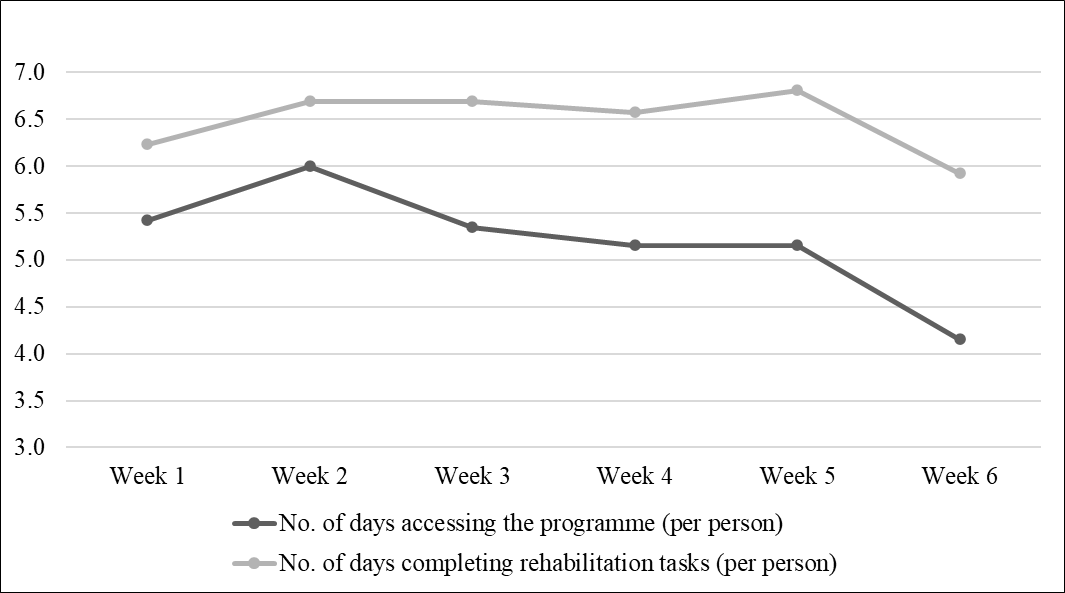


**Figure S1**. Patient engagement in a mobile rehabilitation programme over time (n = 26)
