# Supplementary material for: Patient Engagement in a Mobile App–Based Rehabilitation Program for Total Hip or Knee Arthroplasty: Secondary Data Analysis of a Randomized Controlled Trial
Source: JMIR Mhealth Uhealth. 2024 Oct 1;12:e57635. doi: 10.2196/57635 (PMC11480718; doi:10.2196/57635)
Supplement: Multimedia Appendix 2 [file mhealth_v12i1e57635_app2.pdf]

**CONSORT-EHEALTH Checklist V1.6.1 Report**	**Manuscript Number** **57635**
**(based on CONSORT-EHEALTH V1.6), available at [http://tinyurl.com/consort-ehealth-v1-6].**	
**Date completed**	
10/01/2024 15:28:07	
**by**	
Qingling Wang	
Patient Engagement in a Mobile App–Based Rehabilitation Program for Total Hip or Knee Arthroplasty: Secondary Data Analysis of a Randomized Controlled Trial	

TITLE

1a-i) Identify the mode of delivery in the title

“a mobile app-based rehabilitation program”

1a-ii) Non-web-based components or important co-interventions in title

Not applicable

1a-iii) Primary condition or target group in the title

“total hip or knee arthroplasty”

ABSTRACT

1b-i) Key features/functionalities/components of the intervention and comparator in the METHODS section of the ABSTRACT

"Data were extracted from a pool of 42 participants in the experimental arm of a randomized controlled trial that used a mobile app (WeChat [Tencent Holdings Limited])–based program to support patients’ rehabilitation after total hip or knee arthroplasty."

1b-ii) Level of human involvement in the METHODS section of the ABSTRACT

1b-iii) Open vs. closed, web-based (self-assessment) vs. face-to-face assessments in the METHODS section of the ABSTRACT

1b-iv) RESULTS section in abstract must contain use data

"The participants reported in a rehabilitation diary accessing the program on a mean of 5.2 (SD 2) days per week and completing recommended rehabilitation tasks on a mean of 6.5 (SD 0.8) days per week. The majority (31/42, 74%) posted on the discussion forum, with a mean of 18.1 (SD 21.2) posts. Most participants (37/42, 88%) sent messages to health care professionals, with a mean of 14 (SD 15.9) messages. The program components were visited for a total of 525 times. The program content was read 898 times and shared 82 times in total."

1b-v) CONCLUSIONS/DISCUSSION in abstract for negative trials

INTRODUCTION

2a-i) Problem and the type of system/solution

"This study aimed to investigate patient engagement in a social media app–based rehabilitation program for THA and TKA by analyzing data that had been collected in a randomized controlled trial. The objectives were to (1) investigate patient engagement in the 6-week mobile arthroplasty rehabilitation program through data collected from a written rehabilitation diary, data on the patient’s performance in the program, and data tracked by the app’s background system; and (2) examine the association between patient engagement and their demographic and clinical characteristics.”

2a-ii) Scientific background, rationale: What is known about the (type of) system

"With technological advancement, health care professionals can use information and communication technologies, such as mobile apps, to complement or as an alternative to face-to-face services, to support patients’ rehabilitation after THA and TKA. ... However, more evidence from robust randomized controlled trials based on physical and psychological outcomes is needed to support the implementation of mobile rehabilitation in arthroplasty populations. ... We developed a mobile app (WeChat)-based program that was based on theories and patients’ perceived needs to support patients’ rehabilitation in 6 weeks after THA and TKA. ... However, the extent to which patients were engaged in the mobile app-based rehabilitation program and how this engagement was associated with patients’ characteristics were not investigated.... It is important to understand patient engagement in mobile health interventions because interventions are more efficient and effective when patients are better engaged."

Does your paper address CONSORT subitem 2b?

Please see response to Question 2a-i.

METHODS

3a) CONSORT: Description of trial design (such as parallel, factorial) including allocation ratio

"This study extracted data from the pool of 43 participants in the experimental arm of a single-center, parallel-group, randomized controlled trial conducted at a university hospital in Shanghai, China."

3b) CONSORT: Important changes to methods after trial commencement (such as eligibility criteria), with reasons

Not applicable

3b-i) Bug fixes, Downtimes, Content Changes

Not applicable

4a) CONSORT: Eligibility criteria for participants

"All participants were adults (aged ≥18 years) who (1) were discharged home after a unilateral primary THA or TKA and (2) had access to a 6-week, mobile app–based rehabilitation program after hospital discharge. Patients were excluded from this study if they could not practice recommended rehabilitation exercises due to major complications or serious health conditions such as heart failure or had severe vision impairment."

4a-i) Computer / Internet literacy

4a-ii) Open vs. closed, web-based vs. face-to-face assessments:

This paper is the secondary data analysis of an RCT, and the detailed approach to recruitment has been reported in a previous publication. This has been stated in the main text as “A detailed description of the program has been published [36].”

4a-iii) Information giving during recruitment

4b) CONSORT: Settings and locations where the data were collected

“ at a university hospital in Shanghai, China”

4b-i) Report if outcomes were (self-)assessed through online questionnaires

"The first measured the number of days the participants accessed the program and the number of days the participants completed recommended rehabilitation tasks. These data were obtained by reviewing the participants’ records in their rehabilitation diary.

The second measure counted the number of participants’ posts on the discussion forum and the number of messages sent by the participants to health care professionals.

The third measure focused on the data from the background system of the app, which tracked how many times the content from the program was read and shared by the participants and how often the program components were visited."

4b-ii) Report how institutional affiliations are displayed

5) CONSORT: Describe the interventions for each group with sufficient details to allow replication, including how and when they were actually administered

5-i) Mention names, credential, affiliations of the developers, sponsors, and owners 5-ii) Describe the history/development process

5-iii) Revisions and updating

6-iv) Quality assurance methods

5-v) Ensure replicability by publishing the source code, and/or providing screenshots/screen-capture video, and/or providing flowcharts of the algorithms used

5-vi) Digital preservation

5-vii) Access

"The mobile app–based rehabilitation program was carried out between May 2021 and January 2022. Educational materials such as demonstration videos were uploaded to the app (WeChat) in advance. The participants could learn at home through WeChat installed on their own mobile devices."

5-viii) Mode of delivery, features/functionalities/components of the intervention and comparator, and the theoretical framework

"A 6-week, theory-underpinned rehabilitation program was provided to study participants through the app WeChat, aiming to enhance their self-efficacy during rehabilitation and improve their rehabilitation practice and outcomes. The program provided exercise demonstration videos that were designed for patients after THA and TKA. ... In addition, program components were designed to enhance patients'self-efficacy for rehabilitation, such as setting weekly goals for rehabilitation; scheduling progressive rehabilitation tasks; providing short stories and videos to share the experiences of previous patients; holding a discussion forum that involved health care professionals, patients, and researchers through the app; and providing psychological techniques such as relaxation exercises. To facilitate the participants' learning process, educational materials in the program were designed using visual presentations with short text messages, and a paper booklet on the use of the program was provided in addition to verbal explanations."

5-ix) Describe use parameters

"The participants were suggested to follow the videos and practice for 1 hour per day and at least 5 days per week."

5-x) Clarify the level of human involvement

“holding a discussion forum that involved health care professionals, patients, and researchers through the app”

5-xi) Report any prompts/reminders used

"To improve participants' engagement in the program, the researchers sent an exercise reminder every Monday"

5-xii) Describe any co-interventions (incl. training/support)

Not applicable

6a) CONSORT: Completely defined pre-specified primary and secondary outcome measures, including how and when they were assessed

"The primary outcomes of this study were the number of days the participants accessed the program and the number of days they completed the recommended rehabilitation tasks as recorded in the paper diary. ... Secondary outcomes focused on engagement in a part of the program, including the number of posts made by the participants on the discussion forum, the number of messages they sent to health care professionals through the app, the total number of visits to the program components, and the total number of times the content was read and shared."

6a-i) Online questionnaires: describe if they were validated for online use and apply CHERRIES items to describe how the questionnaires were designed/deployed

6a-ii) Describe whether and how “use” (including intensity of use/dosage) was defined/measured/monitored

Please see response to Question 4b-i.

6a-iii) Describe whether, how, and when qualitative feedback from participants was obtained

6b) CONSORT: Any changes to trial outcomes after the trial commenced, with reasons

7a) CONSORT: How sample size was determined

7a-i) Describe whether and how expected attrition was taken into account when calculating the sample size

Data were extracted from all participants who accessed a mobile app-based arthroplasty rehabilitation program in a randomized controlled trial.

7b) CONSORT: When applicable, explanation of any interim analyses and stopping guidelines

Not applicable

8a) CONSORT: Method used to generate the random allocation sequence

This paper is the secondary data analysis of an RCT, and the detailed approach to randomization has been reported in a previous publication. This has been stated in the main text.

8b) CONSORT: Type of randomisation; details of any restriction (such as blocking and block size)

Please see response to Question 8a.

9) CONSORT: Mechanism used to implement the random allocation sequence (such as sequentially numbered containers), describing any steps taken to conceal the sequence until interventions were assigned

Please see response to Question 8a.

10) CONSORT: Who generated the random allocation sequence, who enrolled participants, and who assigned participants to interventions

This paper is the secondary data analysis of an RCT, and the information about group allocation and participant enrollment has been reported in a previous publication. This has been stated in the main text.

11a) CONSORT: Blinding - If done, who was blinded after assignment to interventions (for example, participants, care providers, those assessing outcomes) and how

11a-i) Specify who was blinded, and who wasn't

This paper is the secondary data analysis of an RCT, and the information about blindness has been reported in a previous publication. This has been stated in the main text.

11a-ii) Discuss e.g., whether participants knew which intervention was the “intervention of interest” and which one was the “comparator”

11b) CONSORT: If relevant, description of the similarity of interventions

Not applicable

12a) CONSORT: Statistical methods used to compare groups for primary and secondary outcomes

"As the outcome variables were categorical data (eg, 2 categories of the number of days accessing the program) or counting data (eg, number of posts and messages), generalized linear models were used to analyze the association between patient engagement and their demographic and clinical characteristics. Pearson correlation analysis was performed for explanatory variables, and pairs of variables with correlation coefficients greater than 0.7 (age and employment as well as comorbidities and long-term use of medication) were discussed within the research team. A decision was made to remove 2 variables, employment an d long-term use of medication, from the model, as they might be due to highly correlated variables. A collinearity diagnostic was conducted for the remaining explanatory variables. The results showed that the tolerance of the variables was greater than 0.2 and the variance inflation factor was less than 5, indicating that there was no significant collinearity between the variables. Therefore, all remaining explanatory variables (ie, age, gender, BMI, residence, cohabitation status, education level, type of surgery, reasons for surgery, comorbidities, self-efficacy, self-reported physical function, severity of pain, levels of anxiety and depression, and health-related quality of life) were included in generalized linear models. Considering the coprimary outcomes, the statistical significance was set as an α level of .025."

12a-i) Imputation techniques to deal with attrition / missing values

Not applicable

12b) CONSORT: Methods for additional analyses, such as subgroup analyses and adjusted analyses

"an exploratory analysis of changes in these data over 6 weeks is provided in Multimedia Appendix 1"

RESULTS

13a) CONSORT: For each group, the numbers of participants who were randomly assigned, received intended treatment, and were analysed for the primary outcome

13b) CONSORT: For each group, losses and exclusions after randomisation, together with reasons

"As 1 patient dropped out from the study within 1 week after group allocation and did not participate in the mobile rehabilitation program, this study included 42 participants in total."

13b-i) Attrition diagram

14a) CONSORT: Dates defining the periods of recruitment and follow-up

"The mobile app–based rehabilitation program was carried out between May 2021 and January 2022."

14a-i) Indicate if critical "secular events" fell into the study period

14b) CONSORT: Why the trial ended or was stopped (early)

15) CONSORT: A table showing baseline demographic and clinical characteristics for each group

15-i) Report demographics associated with digital divide issues

"Women comprised 74% (31/42) of the participants, and the age ranged from 33 to 83 (mean 66.7, SD 10.4) years. The participants had undergone THA (23/42, 55%) or TKA (19/42,45%). Most participants underwent surgery because of osteoarthritis (35/42, 83%), followed by acute fractures (5/42, 12%) and other reasons (2/42, 5%). The majority (30/42, 71%) had chronic diseases, such as hypertension (19/42, 45%) and diabetes mellitus (9/42, 21%), and were taking medication for these diseases. Most participants (39/42, 93%) lived with their families after THA and TKA surgery, and 90% (38/42) of the participants were unemployed or retired at the time of the study. In terms of education levels, 24% (10/42) had completed education at university or college, 31% (13/42) had completed high school education or had equivalent learning experience, 29% (12/42) had completed middle school education, and 17% (7/42) had primary school education or lower.

16a) CONSORT: For each group, number of participants (denominator) included in each analysis and whether the analysis was by original assigned groups

16-i) Report multiple "denominators" and provide definitions

16-ii) Primary analysis should be intent-to-treat

17a) CONSORT: For each primary and secondary outcome, results for each group, and the estimated effect size and its precision (such as 95% confidence interval)

Information reported in Tables 1 and 2.

17a-i) Presentation of process outcomes such as metrics of use and intensity of use

"The majority of study participants (26/42, 62%) returned their completed rehabilitation diary to health care professionals. In the diaries, 65% (17/26) reported accessing the program in 30

or more days, and 96% (25/26) reported completing recommended rehabilitation tasks in 30 or more days. The number of days that the participants accessed the rehabilitation program ranged from 1.3 to 7 (mean 5.2, SD 2; median 5.8, IQR 4.1) days per week, and the number of days that the participants completed recommended rehabilitation tasks ranged from 3.5 to 7 (mean 6.5, SD 0.8; median 7, IQR 0.8) days per week.)."

17b) CONSORT: For binary outcomes, presentation of both absolute and relative effect sizes is recommended

18) CONSORT: Results of any other analyses performed, including subgroup analyses and adjusted analyses, distinguishing pre-specified from exploratory

"an exploratory analysis of changes in these data over 6 weeks is provided in Multimedia Appendix 1"

17-i) Subgroup analysis of comparing only users

19) CONSORT: All important harms or unintended effects in each group

19-i) Include privacy breaches, technical problems

Not applicable.

19-ii) Include qualitative feedback from participants or observations from staff/researchers

DISCUSSION

20) CONSORT: Trial limitations, addressing sources of potential bias, imprecision, multiplicity of analyses

20-i) Typical limitations in ehealth trials

"Engagement data were extracted from 1 arm of a randomized controlled trial. This may have introduced selection bias in the results as patients who voluntarily participated in the trial might be more familiar with technology and thus better engaged in the program. The results of this study should be interpreted with caution, as the sample size was small, which may have reduced the power of the statistical analysis [55]. Due to the privacy policy of WeChat, the engagement data from the app's background system, such as the times of reading and sharing the program content, are global counts, and the data of each participant cannot be identified. This limits the exploration of individual disparities in program usage. The current program is a 6-week mobile rehabilitation program focusing on acute rehabilitation after arthroplasty, which limits the exploration of how patient engagement in mobile rehabilitation changes with recovery after surgery."

21) CONSORT: Generalisability (external validity, applicability) of the trial findings 21-i) Generalizability to other populations

"Patient engagement in mobile arthroplasty rehabilitation is associated with their characteristics, such as education level, cohabitation status, age, type of surgery, presence of comorbidities, and sense of self-efficacy. The future design of mobile app – based arthroplasty rehabilitation programs could consider these characteristics and use strategies, such as involving family members, to improve patient engagement with these interventions.."

21-ii) Discuss if there were elements in the RCT that would be different in a routine application setting

22) CONSORT: Interpretation consistent with results, balancing benefits and harms, and considering other relevant evidence

22-i) Restate study questions and summarize the answers suggested by the data, starting with primary outcomes and process outcomes (use)

"This study investigated patient engagement in a social media app (WeChat)–based arthroplasty rehabilitation program by analyzing data obtained from the app (eg, posts, messages, and reading and sharing of program content) and a written rehabilitation diary (eg, days patients accessed the program and completed recommended rehabilitation tasks). The proportions of patients who accessed the WeChat-based program and who completed recommended rehabilitation tasks are comparable with that reported in previous arthroplasty rehabilitation programs using specifically designed apps or commercial medical apps [7,11,26]. Usage data, such as the number of days per week the patients accessed the program, the average number of posts on the discussion forum, and the average number of messages sent by the patients, are also similar to that in a TKA rehabilitation program based on a commercial medical app [10]. The previous programs were considered to have high levels of patient acceptance and engagement [10,26]. These findings provide evidence that arthroplasty rehabilitation delivered by WeChat has good patient engagement. Furthermore, the findings from this study suggest that patient engagement in mobile arthroplasty rehabilitation is associated with demographic characteristics such as age; education level; and cohabitation status and clinical characteristics such as type of surgery, presence of comorbidities, and sense of self-efficacy. This adds to the body of evidence about the relationship between patient engagement in mobile arthroplasty rehabilitation and personal characteristics."

22-ii) Highlight unanswered new questions, suggest future research

"Future studies could investigate the specific support required by patients after TKA and solutions to address the requirements so that mobile rehabilitation interventions are tailored for this population.

Therefore, age as a predictor of patient engagement in mobile health is inconclusive, and more research is needed to understand the relationship between them and possible mediating factors.

However, further investigation is needed on how to incorporate electronic diaries into social media app–based arthroplasty rehabilitation programs.

OTHER INFORMATION

23) CONSORT: Registration number and name of trial registry

"Australian New Zealand Clinical Trials Registry ACTRN12621000867897; https://tinyurl.com/mtdw25fp."

24) CONSORT: Where the full trial protocol can be accessed, if available

The protocol has been registered at the Australian New Zealand Clinical Trials Registry and published.

25) CONSORT: Sources of funding and other support (such as supply of drugs), role offunders

This study was not funded.

X26-i) Comment on ethics committee approval

x26-ii) Outline informed consent procedures

X26-iii) Safety and security procedures

X27-i) State the relation of the study team towards the system being evaluated
